# A novel gene signature for predicting outcome in colorectal cancer patients based on tumor cell-endothelial cell interaction via single-cell sequencing and machine learning

**DOI:** 10.1016/j.heliyon.2025.e42237

**Published:** 2025-01-24

**Authors:** Lina Pang, Qingxia Sun, Wenyue Wang, Mingjie Song, Ying Wu, Xin Shi, Xiaonan Shi

**Affiliations:** a Department of Oncology, The First Affiliated Hospital of Zhengzhou University, Zhengzhou, Henan, China; b China Medical University, Shenyang, 110001, China; c Department of General Practice, the First Hospital of China Medical University, Shenyang, 110001, China; d Department of Phase I Clinical Trial, the First Hospital of China Medical University, Shenyang, 110001, Liaoning, China; e School of Health Management, Institute of Health Sciences of China Medical University, Shenyang, 110122, China; f School of Mathematics and Information Science, Shandong Technology and Business University, Yantai, 264003, China

**Keywords:** Colorectal cancer, Endothelial cells, Prognosis, Single-cell RNA sequencing, Machine learning

## Abstract

**Background:**

The intricate interactions between malignant cells and endothelial cells (ECs) are crucial in the progress of colorectal cancer (CRC). Identifying molecular signatures associated with this interaction could yield critical prognostic insights and inform personalized therapeutic approaches.

**Methods:**

We conducted an in silico study integrating single-cell RNA sequencing and bulk transcriptome data to characterize the cellular heterogeneity of CRC. Through computational cell interaction analysis facilitated the elucidation of signaling dynamics among cell subpopulations linked to CRC prognosis. Prognostic signatures were developed using various machine learning algorithms based on marker genes linked to the identified cell subpopulations. Immune cell infiltration assessment and gene enrichment analysis were performed to characterize CRC patients stratified by the signature.

**Results:**

Our analysis revealed two distinct cell subgroups, Malignant Cluster01 tumor cells, and Tip-like endothelial cells, showing significant interaction and closely associated with colorectal cancer prognosis. Specifically, Malignant Cluster01 subpopulations primarily served as signal senders, while Tip-like endothelial cells acted as receivers in PARs signaling. The Malignant Cluster01 and Tip-like endothelial cells related machine learning-derived prognostic signature (MTMLDPS), demonstrated potent prognostic capability, effectively predicting colorectal cancer patient outcomes across diverse databases. The colorectal cancer group with a high Malignant Cluster01 and Tip-like endothelial cells related machine learning-derived prognostic signature score exhibited significant associations with invasion, epithelial-mesenchymal transition, and angiogenesis pathways, along with immune cell infiltration.

**Conclusion:**

The Malignant Cluster01 and Tip-like endothelial cells related machine learning-derived prognostic signature holds promise for improving prognostic precision and guiding individual therapeutic strategies in colorectal cancer patients. Moreover, our findings emphasize the importance of considering tumor-endothelial cell interactions in cancer prognosis, providing insights for future therapeutic interventions targeting these interactions.

## Highlights

• Malignant Cluster01 tumor cells and Tip-like ECs show significant interaction and are linked to CRC prognosis.
•Malignant Cluster01 and Tip-like ECs related MTMLDPS demonstrated strong prognostic capability, predicting CRC outcomes.
•High MTMLDPS score in CRC correlates with invasion, EMT, angiogenesis, and immune cell infiltration.

## 1 Introduction

Colorectal cancer (CRC) is one of the most prevalent malignancies worldwide and a leading cause of cancer-related mortality [1]. Although there have been significant advancements in diagnostic and treatment methods [[2], [3], [4], [5]], the prognosis for CRC patients remains suboptimal, particularly in advanced stages. Anti-angiogenic therapy has emerged as promising approaches in the management of metastatic CRC [6]. It works by selectively inhibiting the binding of VEGF to its receptor on endothelial cells (ECs), thus reducing tumor angiogenesis and limiting tumor growth [7,8]. Additionally, by promoting vascular normalization within the tumor, anti-angiogenic agents can modulate the immune microenvironment, leading to enhanced therapeutic effects. The combination of anti-angiogenic therapy with chemotherapy is recommended for first-line, second-line, and third-line treatment of CRC [9,10]. However, despite initial successes, the efficacy of current treatments varies among patients, and treatment resistance remains a significant issue [11]. Resistance to anti-angiogenic agents may arise from EC heterogeneity [12] and the presence of different subtypes of vascular endothelial growth factors [13]. Tumor-derived ECs exhibit varied angiogenic abilities and are accompanied by epithelial-mesenchymal transition (EMT) [14]. Hence, there is a critical demand to identify biomarkers for predicting the prognosis and treatment effects in CRC patients.

Tumor microenvironment (TME) is a multifaceted ecosystem consisting of various cell types, extracellular matrix components, and signaling molecules, all contributing to tumor immune evasion and immunosuppression [15]. Among these components, ECs constitute the inner surface of blood vessels and actively participate in tumor angiogenesis, vascular remodeling, and immune modulation [16]. Current progress in single-cell profiling techniques have revealed the heterogeneity in EC populations, revealing distinct subclusters with unique functional phenotypes [17]. The tumor-specific EC subclusters represent promising targets for novel therapeutic interventions aimed at disrupting tumor angiogenesis and enhancing treatment efficacy [18]. The interaction among specific cancer cells and EC subpopulations is integral to the regulation in key tumorigenic processes, including tumor growth, metastasis, and therapeutic response [[19], [20], [21]]. Deciphering the genes involved in this dynamic interplay holds immense significance for unraveling candidate biomarkers for prognosis and treatment targets.

For this study, we combined bulk RNA sequencing (RNA-seq) data and single-cell RNA sequencing (scRNA-seq) data, and utilized machine learning algorithms to characterize genes related to interaction between tumor cells and ECs. Through the comprehensive analysis, our objective was to develop a prognostic gene signature capable of accurately predicting outcomes in CRC patients, thereby guiding personalized treatment strategies and enhancing patient survival.

## 2 Methods

### 2.1 Data download

The RNA-seq data of 462 colorectal cancer patients, processed with log2 (count + 1), somatic mutation data treated with MuTect2, and associated clinical information were sourced from The Cancer Genome Atlas [22] (TCGA) through the UCSC Xena website [23] (https://xenabrowser.net/datapages/). GSE173839 [24], GSE39582 [25], GSE20916 [26], GSE21510 [27], GSE5206 [28], GSE33113 [29], GSE23878 [30], GSE9348 [31], GSE110224 [32], and GSE144735 [33] (the Catholic University of Leuven dataset contains core and marginal tumor regions and corresponding normal mucosal specimens, derived from 6 Belgium CRC patients, totaling 27,414 cells) were obtained from the Gene Expression Omnibus (GEO) database of the National Center for Biotechnology Information (NCBI) [34] and their clinical data were organized. The clinical data for these datasets were organized, and all patient samples analyzed were confirmed to be Stage M0.

### 2.2 scRNA-seq data quality control and preprocessing

Raw gene expression matrices were processed into Seurat objects using the Seurat R package (V4.0). Cells were filtered into non-immune cells (>40 % mitochondrial genes or 200–6000 genes) and immune cells (>25 % mitochondrial genes or 200–4000 genes). To further remove doublets, the Scrublet pipeline was applied to each batch of scRNA-seq data, with the expected doublet rate set to 0.05. 27,414 cells were obtained for further analysis, including 13,818 non-immune cells and 13,596 immune cells. The gene expression matrix was log-transformed and standardized by the total unique molecular identifier (UMI) count for single cell. To correct for technical and biological differences and improve cell type identification accuracy, canonical correlation analysis (CCA) available in Seurat was applied to all samples prior to identifying cell types. Using the ‵FindVariableFeatures' function in Seurat, 2000 highly variable genes were identified, which were subsequently used for principal component analysis (PCA). The elbow point in the scree plot determined the number of PCs determined the number of principal components (PCs) for different cell types, with the first 20 PCs selected to run FindNeighbors and RunUMAP functions. We clustered the cells by ‵FindClusters' function, exploring resolutions between 0.1 and 1 to optimize cell clustering. A resolution of 0.8 was chosen for clustering. Finally, we used uniform manifold approximation and projection (UMAP) to display the clusters in a two-dimensional space.

### 2.3 GSVA analysis

Gene Set Variation Analysis (GSVA) [35], a non-parametric unsupervised analytical analysis, assesses gene set enrichment by transforming gene expression matrices from different samples into matrices representing gene set expression across samples. This technique evaluates pathway enrichment differences among samples. GSVA analysis was conducted on tumor Hallmark pathways using the "h.all.v7.5.2.symbols" gene set from MSigDB, with default parameters: Gaussian kernel, minimum gene set size of 15, and a maximum of 500 genes per set.

### 2.4 CNV estimation

InferCNV package (V 1.6.0) was utilized to estimate cell CNVs and to identify cancer cells using the default configuration. CNV signals for single cell were estimated using a sliding window of 100 genes. Prior to analysis, genes with an average count below 0.1 across all cells were excluded, and a dynamic cutoff of 1.3 s.d. was applied to denoise the signals.

In order to distinguish malignant cells exhibiting clonal large-scale chronic chromosome copy number variation (CNVs), the R package InferCNV [36] (v 0.99.7) was employed in inferring chromosomal CNVs for each cell. The CNV score for single cells was calculated following the method described by Peng et al. [37]. In the InferCNV algorithm, epithelial cells from the normal group served as the control group, while epithelial cells from the tumor edge and tumor center were used as the analysis group. The calculated CNV scores were standardized and displayed through UMAP maps, and the clustering position relationship between malignant epithelial cells and normal epithelial cells was clarified.

### 2.5 Genetic difference analysis

In GSE144735, the FindAllmarker function in Seurat package was applied to analyze and screen significant genes with differential expression in two types of malignant epithelial cells. We established the thresholds of absolute log2 fold change (log2FC) > 1 and P < 0.05, in accordance with commonly accepted standards for biologically meaningful expression changes. The log2FC threshold prioritizes genes with substantial changes, while the P-value of 0.05 ensures statistical significance, addressing multiple comparisons typical in high-throughput analyses. Up-regulated genes had log2FC > 1 and P < 0.05, while down-regulated genes had log2FC < −1 and P < 0.05. The distinctly expressed genes were visualized using S-maps and heatmaps, and their biological characteristics in various subpopulations of malignant epithelial cells were evaluated by GO enrichment analysis.

### 2.6 Signature generated by integrative machine learning methods

To construct an accurate and stable consensus MTMLDPS, we integrated 101 possible algorithm combinations and 10 machine learning methods, including random survival forest (RSF), CoxBoost, Lasso, Ridge, generalised boosted regression modelling (GBM), elastic network (Enet), survival support vector machine (survival-SVM), stepwise Cox, partial least squares regression for Cox (plsRcox), and supervised principal components (SuperPC).

The process for generating the signature was as follows: (a) Screening genes related to Malignant Cluster01 subpopulation of malignant tumor cells and Tip-like ECs through 1000 Lasso-cox regression models in TCGA-COAD, GSE17537, GSE17536, and GSE39582 datasets. The results of the four datasets were intersected by Upset plots, where genes appearing in two or more datasets were identified as CRC prognostic related genes; (b) Subsequently, to develop prognostic models for the prognostic lncRNAs, 101 algorithm combinations were employed in TCGA_CRC under leave-one-out cross-validation (LOOCV); (c) The performances of each model were evaluated across three prognostic cohorts (GSE39582, GSE17537 and GSE17536); (d) For every model, we computed the Harrell's concordance index (C-index) in every cohorts, regarding the model achieving the greatest mean C-index as the best-performing.

### 2.7 Immune cell infiltration assessment and tumor purity analysis

Using the expression data, immune infiltrating cell scores were calculated for every sample through the R software package IOBR and Cibersort [38] analysis. IOBR is a computational tool [39] commonly used in the study of immune tumor biology.

### 2.8 Gene enrichment analysis

Gene Ontology (GO) annotation [40] is a widely employed approach for extensive functional analysis on genes across various scales and dimensions, generally conducted across three aspects: biological process (BP), cellular component (CC), and molecular function (MF). GO functional annotation was conducted on all markedly differentially expressed genes using the R package ClusterProfiler (v 4.6.0) [41] to determine notably enriched biological functions. The enrichment results were illustrated with the R package GOplot, and the significance cutoff for the analysis was established at p < 0.05. In this study, the selected important gene sets related to immune efficacy and prognosis of CRC were enriched and their biological functions were annotated.

### 2.9 Cellular communication analysis

CellChat(v1.1.3) [42] is a commonly used R package to analyze interactions between cells in a straightforward and comprehensible manner. Here, CellChat was used to systematically deduce and evaluate cell-cell communication networks using scRNA-seq data, and to compare the differences in cell interactions between stromal and malignant cells in cancer tissues. Circle plots were then applied to show the intercellular communication between single-cell subpopulations, and bubble plots were used to count all important ligand pairs during cell-to-cell signaling.

### 2.10 Statistical analysis

We performed all data process and analysis by R software (V 4.1.3). For the comparison of continuous variables among two cohorts, we used the independent Student's t-test for normally distributed variables and Mann-Whitney U test (Wilcoxon rank-sum test) for non-normally distributed variables. Between two groups of categorical variables, Chi-square or Fisher's exact test was employed for evaluating the statistical significance. Survival analysis was conducted with the survival package of R. Kaplan-Meier survival curve was generated to present differences in survival, and Log-rank test was generated to estimate the importance of the time-to-event differences among the two patient groups. Multivariate and univariate Cox analyses were carried out by the survival package, P < 0.05 deemed statistical significance.

## 3 Results

### 3.1 Single cell transcriptome spectrum and cell typing of primary colorectal cancer

**Fig. 1:**
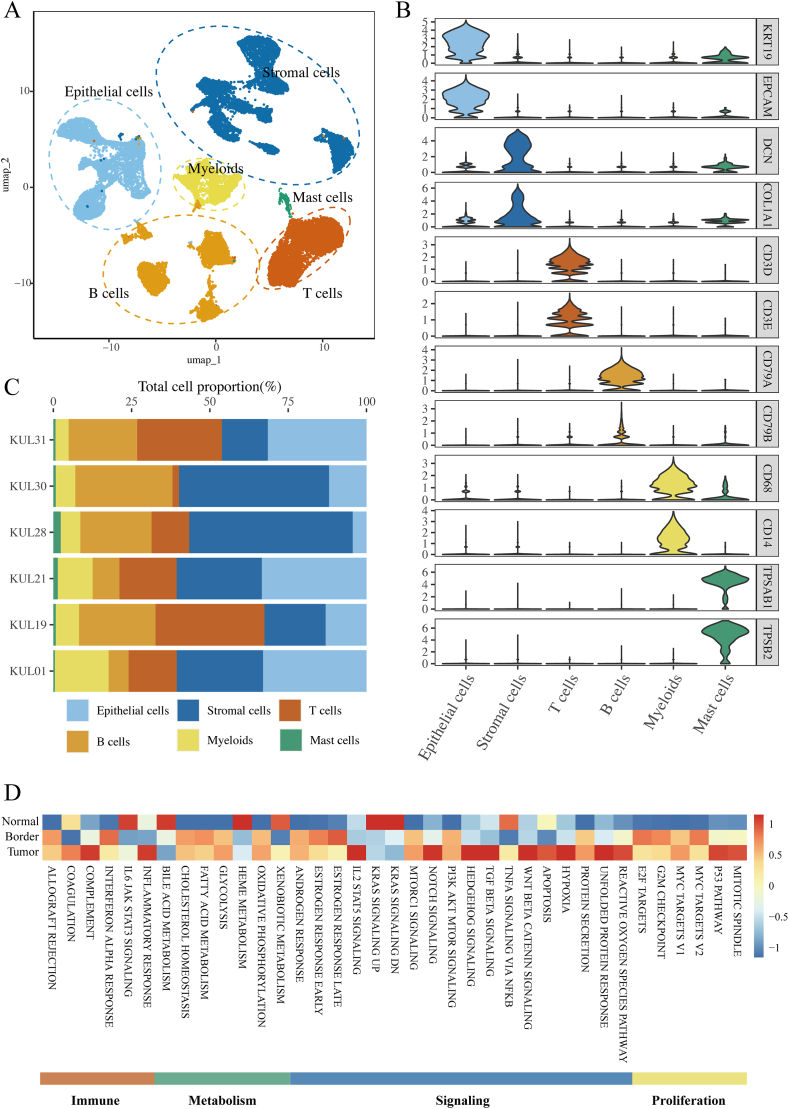
scRNA-seq data analysis in CRC tissues. A. The integrated cell map is visualized in Uniform Manifold Approximation and Projection (UMAP) plot. Cells are shaded according to clusters. B. Violin diagram illustrating marker genes among cell clusters, with color representing the expression of specific genes. C. Distribution of various cell types in different patient samples. D. Heatmap depicting 50 hallmark characteristics of global cell types across adjacent normal tissues, tumor border and tumor core. The intensity of enrichment of different biological functions with different color densities.

In order to comprehensively understand he role of TME in CRC, this study included a publicly available scRNA seq (10X Genomics) dataset GSE144735 containing 6 primary colorectal tumors, with samples from the tumor core region, tumor margin region, and paired adjacent non-cancerous tissues. After multiple quality control steps, we obtained a total of 27414 single-cell transcriptomes from all samples for downstream analysis. These cells were classified into 6 main cell types (Fig. 1A) through R package Seurat function analysis, including 6168 epithelial cells (KRT19, EPCAM, KRT18, KRT8), 7650 stromal cells (DCN, COL1A1, COL1A2, COL3A1, COL5A1), 2676 myeloid cells (CD68, CD14, CD163, CD1C), 5770 T cells (CD3D, CD3E), 4902 B cells (CD79A, CD19), and 248 mast cells (TPSAB1, TPSB2). The expression of the marker genes was shown in Fig. 1B. Next, this study analyzed the proportion of 6 cell types in every patient (Fig. 1C), and the findings revealed that the proportion of various cell types in all patients was comparatively uniform, proving that the sequencing quality and consistency of each sample were good, and the samples had a high proportion of epithelial and stromal cells. To assess the biological features of different tumor regions in CRC samples, this analysis evaluated the normal, tumor, and boundary hallmark characteristics through ssGSEA analysis. The results present that, compared to the tumor margin and adjacent areas, proliferation and signal transduction were significantly enriched in the tumor core region (Fig. 1D).

### 3.2 Tumor tissue-specific Tip-like endothelial cell is a poor prognostic factor for colorectal cancer

**Fig. 2:**
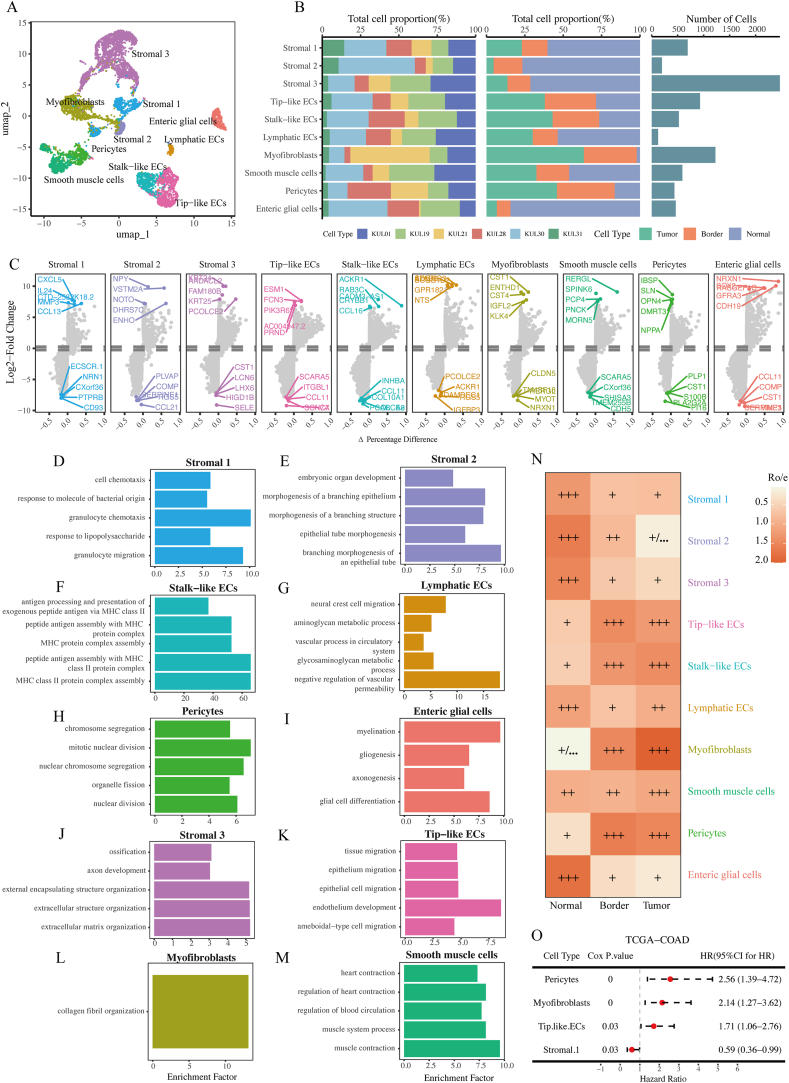
Features of stromal cells in tumor, border and normal tissue. A. UMAP plot of individual stromal cells. B. The bar plots show the proportions of 10 stromal cell types across various samples (left panel) and tissues (middle panel), as well as the total cell quantity per cell type (right panel). C. Representative marker genes across stromal cell types are shown in Dot plot. D-M. Biological functional annotation of stromal cell type. N. Using Cox regression analysis, the prevalence of each stromal cluster across tissues was estimated using the Ro/e score. O. Using Cox regression analysis, the relationship between patient survival and relative cell abundance (estimated through CIBERSORTx) was assessed in TCGA- COAD cohort (n = 462), using two-sided log-rank test to determine P value.

We identified 10 subtypes of stromal cells in patients, namely Stromal 1, Stromal 2, Stromal 3, Tip-like ECs, Stalk-like ECs, lymphoid ECs, myofibroblasts, smooth muscle cells, pericytes and enteric glial cells (Fig. 2A). Although all subtypes of stromal cells were present in all 6 samples (Fig. 2B left panel), it could be observed from Fig. 2B that different subgroups of stromal cells had certain sample area preferences, and there were also certain differences in the number of cells among them (Fig. 2B right panel). The results showed that Stromal 3 cells had the greatest cell count and lymphoid endothelial cells had the least cell count. The proportion of intestinal glial cells, Stromal 1, Stromal 2, and Stromal 3 in adjacent non-cancerous tissues was higher. The genes with differential expression of various cells were shown in Fig. 2C. For example, Tip-like ECs upregulated genes such as ESM1, FCN3, PIK3R6, PRND, and downregulated genes such as SCARA5, ITGBL1, CCL11, etc. Fig. 2D–M showed the functional enrichment of 10 cell types. Tip-like ECs significantly enriched gene pathways such as tissue migration, epithelial cell migration, endothelium development, and amoeboid cell migration. Compared with normal tissues, the tissue characteristics of Tip-like ECs and Stalk-like ECs in tumors tended to show a significantly higher proportion in tumor samples (Fig. 2N). Meanwhile, the proportion of Myofibroblasts and Pericytes in tumor samples also showed a significant increase. The validation analysis in the TCGA-COAD dataset suggested that the high infiltration status of Tip-like ECs was related to unfavorable outcomes of CRC among various cell types (Fig. 2O). Based on the existing research [43] and results above, Tip-like ECs had important biological functions in tumor progression. Focusing on this subtype can better reveal its impact on CRC prognosis.

### 3.3 Malignant Cluster01 in CRC correlates with unfavorable prognosis

**Fig. 3:**
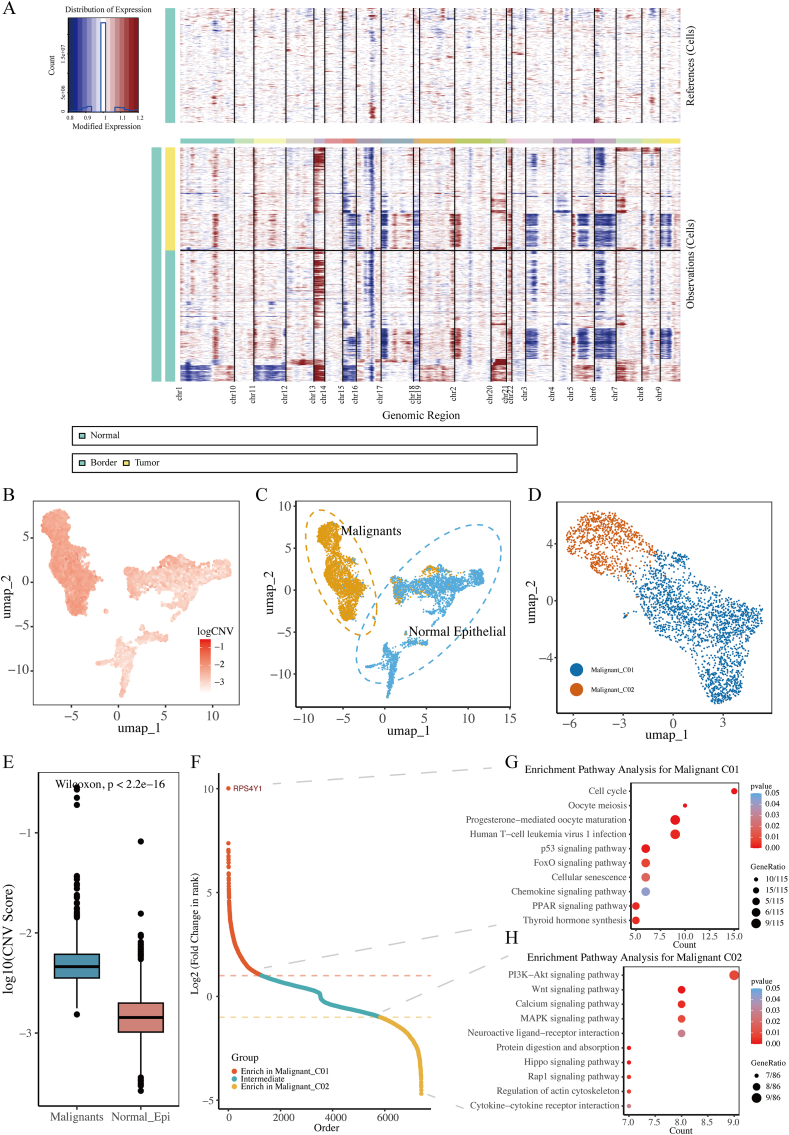
Identification of two malignant cell states. A. Epithelial cells exhibited more prominent CNVs across chromosomes compared to non-epithelial cells. B. UMAP maps of copy number variations (CNVs). C. UMAP maps of normal and malignant epithelial cells. D. UMAP maps of malignant cell states. E. Malignant cells exhibited higher CNV scores. F. S plot of differentially expressed genes between two malignant cell states. G. Enriched pathways in Malignant Cluster01 cells. H. Enriched pathways in Malignant Cluster02 cells.

**Fig. 4:**
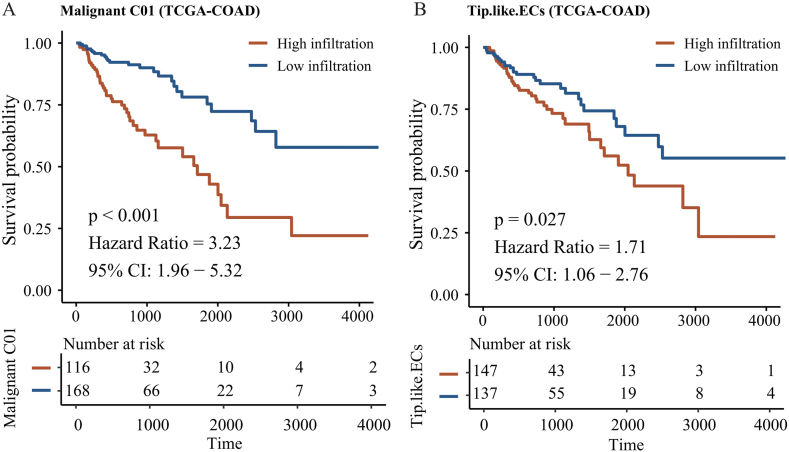
Malignant Cluster01 cells and Tip-like ECs are both poor prognostic predictors. A. In TCGA-COAD patients, Kaplan-Meier (KM) curve displaying that a highly infiltrating Malignant Cluster01 cells was linked to poor OS. B. KM curve displaying that a highly infiltrating Tip-like ECs was linked to poor OS in TCGA-COAD patients.

Copy number inference analysis of all single cells revealed that tumor cells originated from epithelial cells exhibiting notable copy number variations (CNVs) (Fig. 3A). Almost all chromosomes 13, 2, 20, 21, and 7 had been amplified, while chromosomes 6, 9, and 3 were found to be missing. The standardized copy number scores of each epithelial cell were displayed using the UMAP plot (Fig. 3B), and it was found that malignant tumor cells exhibited higher CNV scores with P < 0.001, indicating the statistically significant difference (Fig. 3E). The UMAP diagram displays the arrangement of non-malignant and malignant tumor cells (Fig. 3C). Next, the malignant tumor cells were clustered using R package Seurat, and it was found that they could be divided into two categories: the CRC malignant Cluster01 cell subgroup and the CRC malignant Cluster02 cell subgroup (Fig. 3D). Malignant tumor cells in varying states had been observed to engage in distinct regulatory functions. Fig. 3F showed the differential genes between two malignant tumor cell subtypes, and the subsequent GO enrichment analysis elucidated the distinct biological characteristics of these subtypes. The findings indicated that the subpopulation of Malignant Cluster01 cells in CRC was mainly related to p53 signaling pathway, Oocyte meiosis, and Cell cycle, which mainly reflected the characteristics of unlimited proliferation of tumor cells (Fig. 3G). The subpopulation of Malignant Cluster02 cells in CRC malignant tumors was mainly linked to the PI3K-Akt, Wnt, and calcium signaling pathway, which mainly reflected the characteristics of malignant cell invasion and metastasis (Fig. 3H). Finally, in the analysis of TCGA-COAD dataset, it was found that the high infiltration status of Malignant Cluster01 cell subpopulation (HR = 3.23, P < 0.001, Fig. 4A) and Tip-like EC subpopulation (HR = 1.71, P = 0.027, Fig. 4B) in malignant tumors of CRC patients suggested a poor prognosis. At the same time, multivariate Cox regression analysis indicated that the elevated infiltration state of both Malignant Cluster01 and Tip-like endothelial cells (HR = 1.9581, 95%CI: 1.413–2.713, P < 0.001, Supplementary Table 1) served the role of independent prognostic indicator in CRC patients.

### 3.4 The subsets of Malignant Cluster01 cells and Tip-like ECs in colorectal cancer show a positive relationship with tumor infiltration

**Fig. 5:**
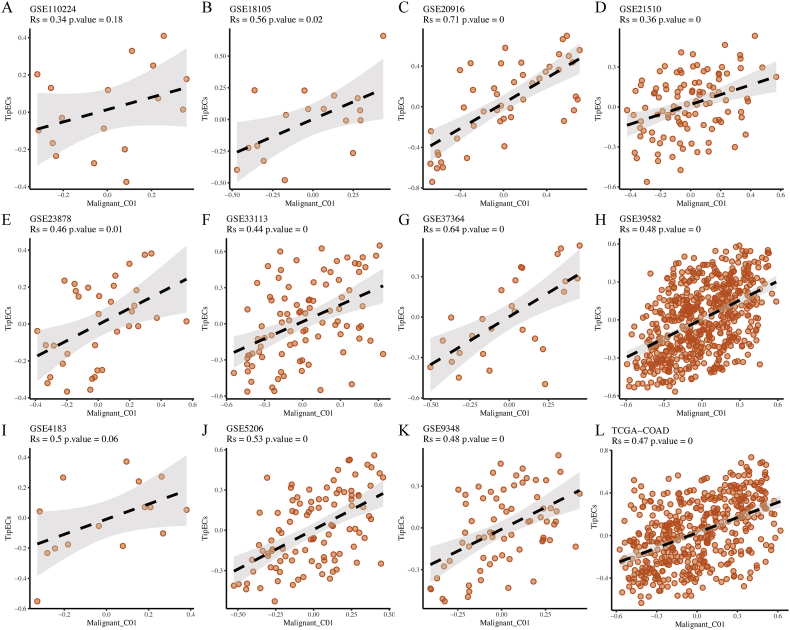
Malignant Cluster01 and Tip-like ECs are positively correlated in tumor infiltration. Scatter plots illustrate the relationship between Malignant Cluster01 and Tip-like ECs infiltration in 12 independent datasets of CRC, such as A. GSE11024; B. GSE18105; C. GSE20916; D. GSE21510; E. GSE23878; F. GSE33113; G. GSE37364; H. GSE39582; I. GSE4183; J. GSE5206; K. GSE9348; L. TCGA-COAD. The error band indicates 95 % confidence interval.

Due to the restricted sample quantity in scRNA-seq cohort, we used the CIBERSORTx method for deconvolution to infer the infiltration of particular cell types in 11 separate public datasets of primary CRC (from GEO database) and TCGA-COAD cohort, and analyzed the correlation between tumor infiltration of two specific cell subpopulations through Spearman correlation analysis. The results showed that in all 12 transcriptional queues of CRC, except GSE110224, the Malignant Cluster01 cell subgroup was positively correlated with the Tip-like EC subgroup (false detection rate [FDR]<0.05 and Spearman correlation coefficient [|Rs|]>0.3 were considered significant correlations, as shown in Fig. 5A–L). The Spearman correlation coefficient ranged from 0.34 in the GSE110224 dataset to 0.71 in the GSE20916 dataset (Fig. 5A–C). This finding emphasized the importance of Malignant Cluster01 and Tip-like endothelial cells in tumor infiltration and the prognosis of patients.

### 3.5 Cell-cell interactions between tumor cell subpopulations Malignant Cluster01 and Tip-like ECs

**Fig. 6:**
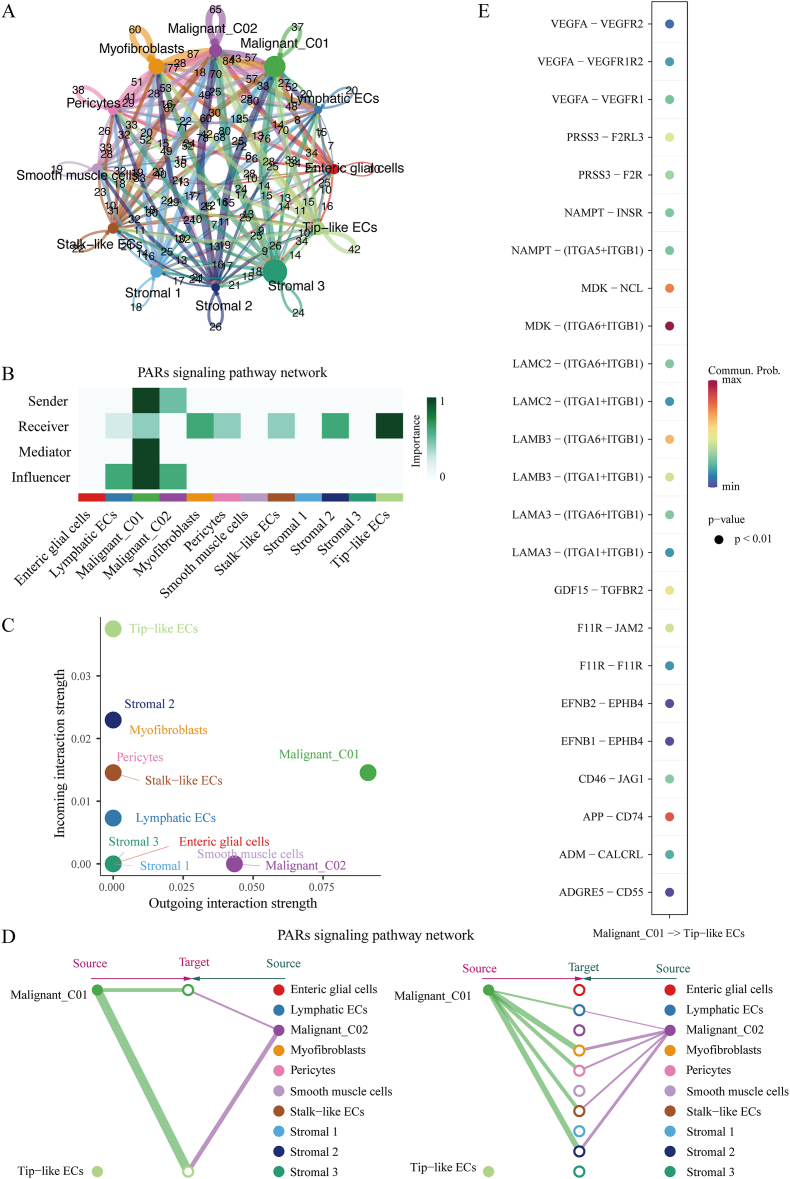
The regulatory network of Tip-like ECs identifies tumor-specifc signaling pathways in CRC. A. Circle plots illustrate the interaction counts among malignant and stromal cells in tumor samples, with broader arrows indicating higher numbers of interactions. B. Heatmap displays the key senders, mediators, receivers, and influencers in PARs signaling in tumor, based on network centrality scores. C. Scatter plot depicting the strength of incoming and outgoing interactions of malignant and stromal cell populations. D. Hierarchical plot illustrating the cell-cell communication network for the PARs. E. Bubble diagram of the interaction between Malignant Cluster01 and Tip-like EC ligand receptor pairs. The graph shows the strongest communication through MDK - (ITGA6+ITGB1).

Cellchat was utilized to investigate the communication characteristics between cells by identifying molecular interactions and ligand-receptor pairs across different cell types. The Count of interactions within the intercellular communication network was shown in Fig. 6A. The findings indicated that cellular interactions mediated by ligand-receptor pairs mainly existed in PARs signaling pathway. Malignant Cluster01 mainly played a role in sending, mediating, and influencing the PARs signaling pathway; Tip-like ECs mainly played a receiving role (Fig. 6B). In the PARs signaling pathway, the incoming signal of Tip-like ECs was strong, and the outgoing signal of Malignant Cluster01 was strong (Fig. 6C). Specifically, the PARs pathway exhibited highly abundant signaling interactions in the subpopulations of Malignant Cluster01 and Tips-like ECs. The network centrality analysis of the PARs pathway showed that Malignant Cluster01 was the main source of PARs ligands targeting the Tip-like EC subpopulation in malignant tumor cells, indicating that the main pathway through PARs interaction was paracrine, as shown in Fig. 6D. MDK - (ITGA6+ITGB1) had the greatest contribution to this pathway (Fig. 6E).

### 3.6 Construction and Verification of MTAIDPS prediction system

**Fig. 7:**
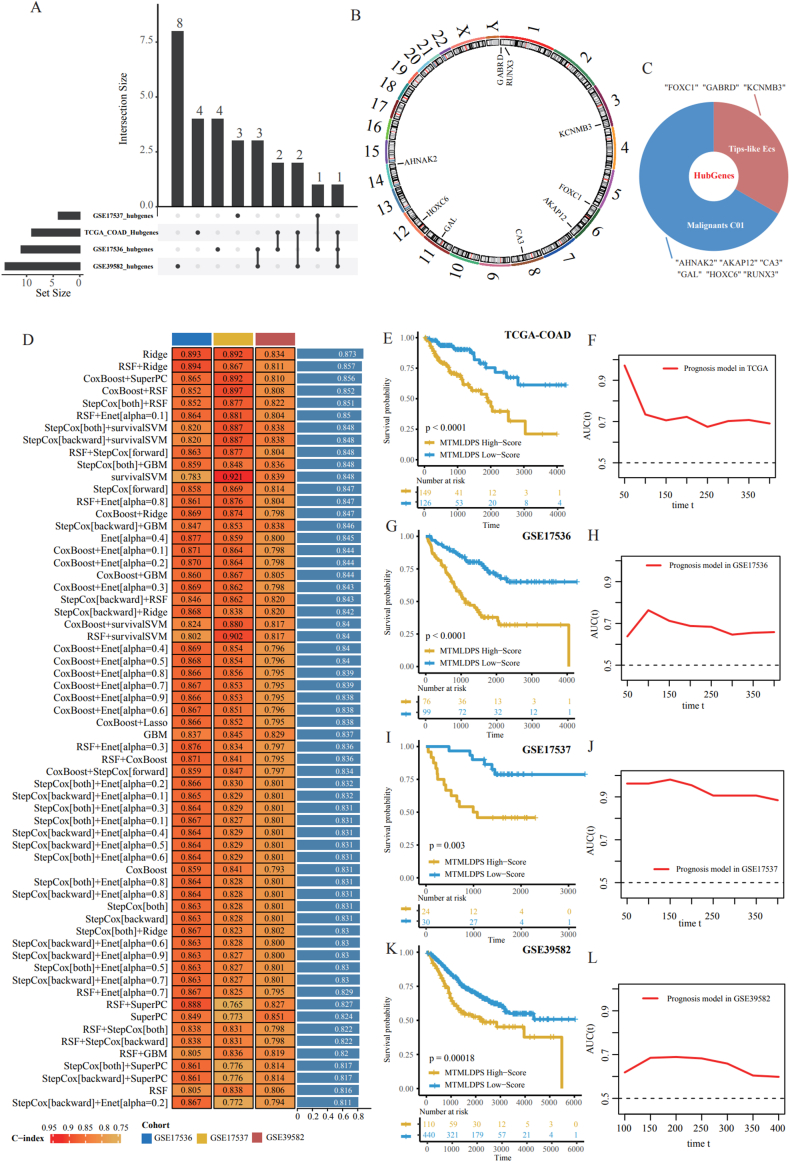
Prognostic value of the MTMLDPS. A. Upset plot of prognostic core genes related Malignant Cluster01 and Tip-like ECs in 4 independent CRC cohorts. B. The position of hub genes on chromosomes. C. The C-index of MTMLDPS and 50 machine learning-derived models in GSE17537, TCGA-COAD, GSE17536 and GSE39582. KM curves of MTMLDPS regarding OS in TCGA-COAD (E), GSE17536 (G), GSE17537 (I), GSE39582 (K). Time-dependent ROC curves of MTMLDPS regarding OS in TCGA-COAD (F), GSE17536 (H), GSE17537 (J), GSE39582 (L).

Prognostic-related genes were screened by the univariate Cox regression model in GSE39582, GSE17537, GSE17536 and TCGA-COAD datasets, followed by 1000 iterations of Lasso Cox analysis to perform dimensionality reduction on the aforementioned genes. The analysis results of four datasets were presented through an Upset plot (Fig. 7A). This study screened for genes that appeared in two or more datasets as prognostic core genes, namely GABRD, RUNX3, KCNMB3, FOXC1, AKAP12, CA3, GAL, HOXC6, and AHNAK2. Fig. 7B showed the positions of the nine prognostic core genes mentioned above on chromosomes. The results showed that GABRD and RUNX3 were on chromosome 1, KCNMB3 was on chromosome 3, FOXC1 and AKAP12 were on chromosome 6, CA3 was on chromosome 8, GAL was on chromosome 11, HOXC6 was on chromosome 12, and AHNAK2 was on chromosome 14. Among them, FOXC1, GABRD and KCNMB3 were related to Tip-like ECs, AHNAK2, AKAP12, CA3, GAL HOXC6 and RUNX3 were associated with Malignant Cluster01, as shown in Fig. 7C. To enhance the prediction of overall survival (OS) in CRC patients, this study used TCGA-COAD dataset as the training set, along with GSE39582, GSE17537 and GSE17536 as the validation sets, to construct MTMLDPS (Malignant Cluster01 and Tip-like ECs related machine learning-derived prognostic signature) with above core genes using various machine learning algorithms, and the consistency index (C-index) of different machine learning algorithms was calculated. The findings indicated that the prediction accuracy of MTMLDPS, developed using different algorithms, ranged from 0.873 for Ridge algorithm to 0.811 for StepCox [back]+Enet [alpha = 0.2], both exceeding 0.75, indicating that MTAIDPS had relatively high prediction ability (Fig. 7D). We validated the MTMLDPS using Kaplan-Meier curves in the four datasets mentioned above, and validated its predictive ability for patient OS over time using time-dependent ROC curves. The findings indicated that MTMLDPS was linked to patient OS in TCGA-COAD (Fig. 7E and F), GSE17536 (Fig. 7G and H), GSE17537 (Fig. 7I and J), and GSE39582 (Fig. 7K and L), with higher MTMLDPS scores indicating poor prognosis. At the same time, our signature was mainly from the perspective of ECs, making it more biologically interpretable than others.

### 3.7 High MTMLDPS score exhibited significant associations with epithelial-mesenchymal transition, invasion, immune cell infiltration and angiogenesis pathways

**Fig. 8:**
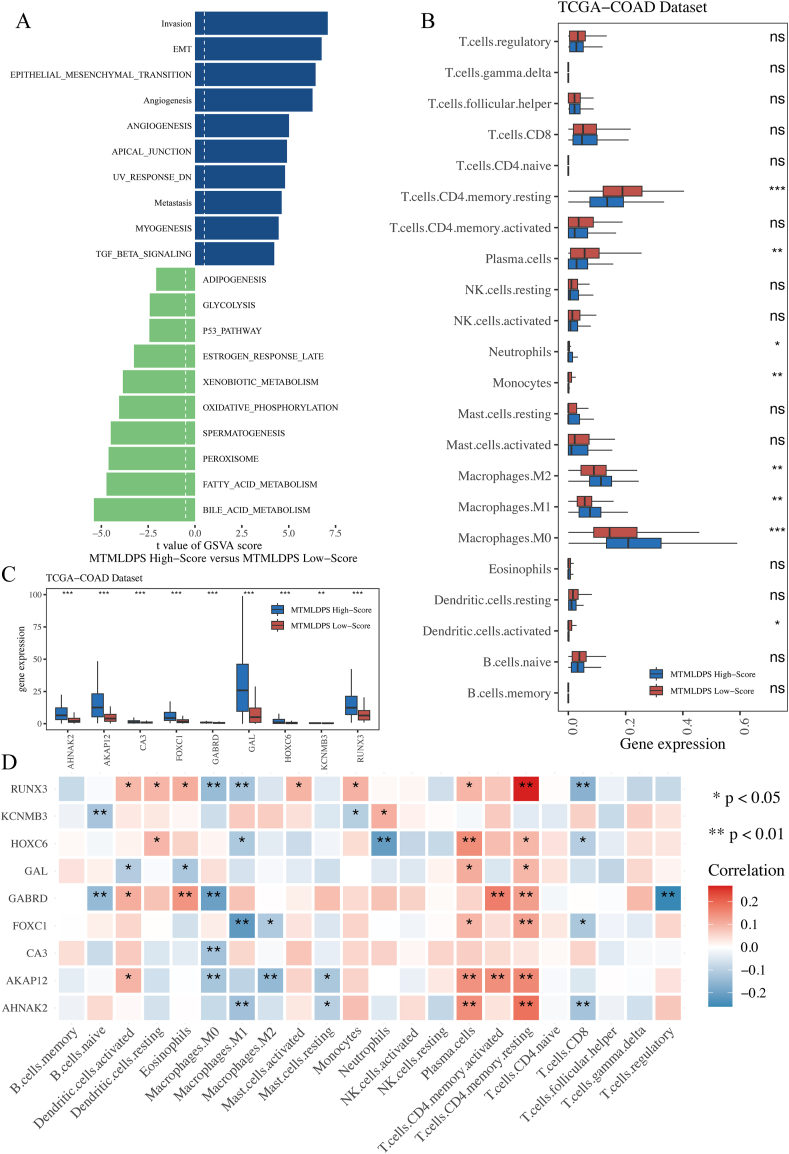
The characteristics of colorectal cancer patients stratified by MTMLDPS score. A. The difference in Hallmark pathway between two patient groups, with blue indicating high enrichment of the pathway in MTMLDPS High-Score group and green indicating high enrichment of the pathway in the MTMLDPS Low-Score group. B. Comparison of immune cell infiltration levels among MTMLDPS High-Score and Low-Score cohorts for CRC patients. C. Comparison of expression levels of 9 core genes among patients from MTMLDPS High-Score and Low-Score groups. D. The correlation between 9 core genes and the infiltration degree of immune cell, with red for positive association, blue for negative association, and ∗ P < 0.05, ∗∗P < 0.01.

The above analysis screened 9 core genes associated with CRC prognosis and construct the MTMLDPS prediction system. TCGA-COAD cohort was thus classified into MTMLDPS Low-Score group and MTMLDPS High-Score group. The differences in 50 important signaling pathways related to tumor hallmarks between the two groups were evaluated. The findings displayed that Invasion, EMT and Angiogenesis pathways were particularly enriched in MTMLDPS High-Score group, while the BILE_ACID_METABOLISM, FATTY_ACID_METABOLISM and PEROXISOME pathways were particularly enriched in MTMLDPS Low-Score group (Fig. 8A). Next, in TCGA-COAD dataset, Cibersort analysis was conducted to evaluate different immune cell infiltration. The findings revealed higher infiltration levels of Macrophage M0 cells, Macrophage M1 cells and Macrophage M2 cells in MTMLDPS High-Score group (p < 0.01), while T.cells.CD4.memory.resting cells and Plasma.cells showed higher levels of infiltration in MTMLDPS Low-Score patients (p < 0.05, Fig. 8B). This study evaluated the expression differences of 9 core genes across different MTMLDPS groups, revealing that, as opposed to MTMLDPS Low-Score group, these genes exhibited higher expression in MTMLDPS High-Score cohort (p < 0.05, Fig. 8C). Ultimately, Fig. 8D showed the relationship among the expression of 9 important prognostic genes and various infiltration of immune cell in TCGA-COAD, revealing that T.cells.CD4.memory.resting cells were positively linked to RUNX3, HOXC6, GAL, GABRD, FOXC1, AKAP12, and AHNAK2 (P < 0.05), while Macrophage M0 was negatively correlated with RUNX3, GABRD, CA3, and AKAP12 (P < 0.05).

## 4 Discussion

The intricate crosstalk between tumor cells and specific ECs is essential for tumor growth, metastasis, and therapeutic resistance [[19], [20], [21]]. In this study, through scRNA-seq analysis, we identified two cell subgroups both associated with colon cancer prognosis: tumor cell subgroup Malignant Cluster01 and EC subgroup Tip-like ECs. Cell-cell communication analysis indicated that Malignant Cluster01 subpopulations primarily act as signal senders, while Tip-like ECs function as receivers in PARs signaling. A novel prognostic prediction system named MTMLDPS based on marker genes associated with these cell subgroups was developed for CRC patients using machine learning algorithms. The signature showed associations with pathways related to invasion, EMT, and angiogenesis. Additionally, it correlated with the infiltration of immune cell, comprising resting memory CD4^+^ T cells and Macrophages.M0.

In comparison with traditional RNA sequencing, scRNA-seq technology allows scholars to assess the heterogenicity of tumor and stromal cells at cellular level, distinguishing the gene expression profiles across different cell types and thereby identifying specific genes for each cell [[44], [45], [46]]. By Unifying bulk and single-cell RNA-seq data, we observed Tip-like ECs as particularly present within tumor samples, suggesting their role as therapeutic and prognostic targets in CRC. Tip-like ECs are crucial in sprouting angiogenesis and endothelial migration [43], potentially fostering resistance to anti-angiogenic therapies and tumor angiogenesis through interactions with other cell types. Notably, we found a significant association between Tip-like ECs and Malignant Cluster01 subpopulations of CRC cells, both of which are linked to inferior CRC prognosis. This implied their collaborative role in inhibiting anti-tumor immune responses and enhancing tumor angiogenesis. We also identified genes serving as specific markers for both Tip-like ECs and Malignant Cluster01 tumor cells, supporting their utility in prognostic and therapeutic strategies for CRC patients.

Furthermore, using cell-cell communication analysis, we identified the potential pathways underlying the interaction between the Tip-like ECs and the identified malignant epithelial cell subpopulations. Malignant Cluster01 tumor cells were primarily identified as signal senders within the PARs signaling pathway, which has been connected to the promotion of cancer growth, invasion, and metastasis. Our observations further indicate that Tip-like ECs may actively respond to PARs signaling cues from Malignant Cluster01 tumor cells, particularly serving as receivers via the MDK-(ITGA6+ITGB1) ligand-receptor pairs. Previous studies have implicated MDK (Midkine) in promoting tumor angiogenesis and metastasis in various cancers [47,48]. Moreover, the integrin complex ITGA6+ITGB1 has been correlated with tumor cell adhesion, invasion, and migration [49]. Therefore, the association between malignant cells and ECs within the PARs signaling pathway, facilitated by MDK and the ITGA6+ITGB1 integrin complex, might significantly contribute to the invasion and metastasis in CRC cells. By identifying these intricate interactions, our study offers valuable perspectives for future research targeting tumor cell-endothelial cell crosstalk.

Researchers have integrated various machine learning techniques to pinpoint key genes in different tumor type, leading to the development of more precise prognostic models [50]. Taking into account the significant predictive value and the strong association between Tip-like ECs and Malignant Cluster01 cells, this study further employed multiple machine learning methods to develop a novel MTMLDPS scoring system for CRC patients, utilizing the genes implicated in tumor cell-endothelial cell interaction. The MTMLDPS consisted of 6 marker genes of Malignant Cluster01 cells (AHNAK2, AKAP12, CA3, GAL, HOXC6, and RUNX3) and 3 marker genes of Tip-like ECs (FOXC1, GABRD, and KCNMB3). It consistently exhibited high prognostic accuracy, with C-index values ranging from approximately 0.811 to 0.873 across various algorithms. This robust performance highlights the reliability and effectiveness of the scoring system in forecasting CRC outcomes. Furthermore, the time-dependent ROC analysis conducted across different independent validation cohorts, including GSE17536, GSE17537, and GSE39582, revealed high and stable performance. Kaplan-Meier analysis also showed unfavorable outcomes among patients with high scores across all cohorts. These findings highlight the clinical utility and potential application value of the MTMLDPS in predicting prognosis and guiding personalized treatment strategies for CRC patients. Clinicians can leverage this signature to identify individuals who are expected to respond well to more intensive therapeutic approaches or closer monitoring, particularly those exhibiting high MTMLDPS scores.

Based on the scoring of the MTMLDPS, we further divided CRC patients into two groups. The group with higher malignant scores exhibited enrichment in pathways associated with invasion, EMT, and angiogenesis. These findings are consistent with earlier research emphasizing the crucial function of ECs in promoting tumor invasion and angiogenesis [51,52]. Factors secreted by ECs, including VEGF, matrix metalloproteinases (MMPs) and angiopoietin-2 (Ang-2), have been shown to promote angiogenesis and EMT in various cancer types, facilitating tumor cell invasion and metastasis [[53], [54], [55]]. It suggests that this signature could function as an important biomarker for forecasting tumor behavior and response to treatment. In clinical practice, integrating the MTMLDPS into routine assessments could facilitate personalized treatment plans, enabling targeted therapies that address the unique tumor microenvironment. Additionally, compared to MTMLDPS Low-score group, our analysis of immune cells revealed significantly higher proportion of Macrophages.M0 and lower resting memory CD4^+^ T cells in MTMLDPS High-Score group. Recent studies have suggested that tumor ECs may facilitate cancer cell evasion from immune surveillance and contribute to cancer progression [56,57]. The interaction between macrophages and ECs could regulate tumor inflammation and metastasis [58], and malignant ECs, in the cancer microenvironment, have been shown to inhibit CD4^+^ T cell activity [59]. Our findings align with previous studies, suggesting a complex communication network among tumor cells, ECs, and immune cells [60], which may help develop potential treatment targets and contribute to future applications. As the field moves toward more personalized approaches, the insights gained from our findings may help clinicians make informed decisions about combining anti-angiogenic therapies with immunotherapies, ultimately improving patient outcomes in CRC.

Nevertheless, this study has certain limitations. Firstly, it relied on retrospective data from public databases to construct the risk signature, highlighting the critical need for validation through future prospective studies involving multicenter CRC cohorts. Such population-based validation is essential to confirm the clinical applicability of our findings. Secondly, all patient samples analyzed in our study were from M0 stage patients, and further validation is needed to determine their performance in more advanced stages. Additionally, while the study emphasized the possible prognostic significance of the risk signature, further laboratory investigations both in vitro and in vivo should be conducted to elucidate the exact role in CRC development and progression. Finally, the reliance on RNA sequencing poses challenges in clinical practice, as this method is not commonly performed for patients, underscoring the need for more accessible diagnostic tools in the future.

To conclude, through the integration scRNA-seq and bulk transcriptome data, we recognized two distinct cell subgroups with significant interaction, Malignant Cluster01 tumor cells and Tip-like ECs, both closely linked to CRC prognosis. Furthermore, utilizing machine learning techniques, we constructed a novel prognostic prediction system, MTMLDPS, on the basis of marker genes associated with the two cell subpopulations, which has enormous potency to distinguish the outcome of CRC patients. Additionally, we observed correlations between the MTMLDPS and immune cell infiltration, further emphasizing its clinical relevance. Given the critical role of tumor-endothelial cell interactions in cancer progression, our findings suggest several therapeutic strategies, such as targeting specific signaling pathways involved in these interactions or employing immunotherapeutic approaches that enhance the immune response to tumor-endothelial communication. These insights may lay the foundation for personalized treatment approaches that aim to disrupt these interactions, with the primary aim of enhancing patient prognosis and treatment outcomes.

## 5 Conclusions

The Malignant Cluster01 and Tip-like endothelial cells related machine learning-derived prognostic signature holds promise for improving prognostic accuracy and guiding personalized treatment strategies in colorectal cancer patients. Moreover, our findings emphasize the importance of considering tumor-endothelial cell interactions in cancer prognosis, providing insights for future therapeutic interventions targeting these interactions.

## CRediT authorship contribution statement

**Lina Pang:** Writing – original draft, Project administration, Conceptualization. **Qingxia Sun:** Writing – original draft, Formal analysis. **Wenyue Wang:** Visualization, Methodology, Writing – review & editing. **Mingjie Song:** Formal analysis, Data curation. **Ying Wu:** Methodology, Supervision. **Xin Shi:** Conceptualization, Methodology. **Xiaonan Shi:** Writing – review & editing, Funding acquisition, Conceptualization, Supervision.

## Ethical Statement

All data were publicly obtained and can be accessed from the TCGA (https://portal.gdc.cancer.gov/) and GEO databases (https://www.ncbi.nlm.nih.gov/geo/). The Institutional Review Boards of the First Affiliated Hospital of Zhengzhou University and the First Hospital of China Medical University deemed ethical approval unnecessary.

## Consent for publication

All authors have given their consent for publication of this manuscript.

## Data availability statement

The data generated and analyzed during the study are available in the TCGA (https://portal.gdc.cancer.gov/) and GEO databases (https://www.ncbi.nlm.nih.gov/geo/), under the public repository accession number GSE173839, GSE39582, GSE20916, GSE21510, GSE5206, GSE33113, GSE23878, GSE9348, GSE110224, and GSE144735.

**Table:** Abbreviations

AUC	area under the curve
BP	biological process
CC	cellular component
CNVs	copy number variation
CRC	colorectal cancer
ECs	endothelial cells
EMT	epithelial-mesenchymal transition
GEO	Gene Expression Omnibus
GO	Gene Ontology
GSVA	Gene Set Variation Analysis
MF	molecular function
ML	Machine Learning
MMPs	matrix metalloproteinases
MTMLDPS	Malignant Cluster01 and Tip-like ECs related machine learning-derived prognostic signature
NCBI	National Center for Biotechnology Information
PCA	Principal Component Analysis
RNA-seq	bulk RNA sequencing
scRNA-seq	single-cell RNA sequencing
TCGA	The Cancer Genome Atlas
TME	tumor microenvironment
UMAP	Uniform Manifold Approximation and Projection
UMI	unique molecular identifier

## Funding

This work was funded by Henan Provincial Medical Science and Technology Research Program Joint Construction Project (LHGJ20190024).

## Declaration of competing interest

The authors declare that they have no known competing financial interests or personal relationships that could have appeared to influence the work reported in this paper.
