# Supplementary material for: A novel gene signature for predicting outcome in colorectal cancer patients based on tumor cell-endothelial cell interaction via single-cell sequencing and machine learning
Source: Heliyon. 2025 Jan 24;11(3):e42237. doi: 10.1016/j.heliyon.2025.e42237 (PMC11815678; doi:10.1016/j.heliyon.2025.e42237)
Supplement: Multimedia component 1 [file mmc1.docx]

**Supplementary Table 1**

|  | coef | HR | se(coef) | z | P | exp(coef) | exp(-coef) | 95%CI |
| --- | --- | --- | --- | --- | --- | --- | --- | --- |
| Gender Male | 0.1905 | 1.2098 | 0.2494 | 0.764 | 0.44500 | 1.210 | 0.8265 | 0.742—1.973 |
| Stage 1 | 0.8640 | 2.3727 | 0.2852 | 3.030 | 0.00245 ** | 2.373 | 0.4215 | 1.357—4.150 |
| mt | 0.6720 | 1.9581 | 0.1664 | 4.039 | 5.37e-05 *** | 1.958 | 0.5107 | 1.413—2.713 |

The variable "mt" represents the cumulative score of two cell types: Malignant Cluster01 and Tip-like endothelial cells. Each of these cell types contributes a score of 1, indicating high infiltration.
